# Mapping the applications and methodological characteristics of the TreeScan method in pharmacovigilance and beyond: a scoping review of 44 studies

**DOI:** 10.3389/fphar.2026.1905426

**Published:** 2026-07-24

**Authors:** Hailong Li, Ruonan Gao, Jianing Liu, Yuanhui Hu, Linan Zeng, Xuefeng Jiao, Zhenyan Bo, Sha Diao, Siyan Zhan, Lingli Zhang

**Affiliations:** 1 Department of Pharmacy/Evidence-Based Pharmacy Center, West China Second University Hospital, Sichuan University, Chengdu, China; 2 Children’s Medicine Key Laboratory of Sichuan Province, Chengdu, China; 3 NMPA Key Laboratory for Technical Research on Drug Products In Vitro and In Vivo Correlation, Chengdu, China; 4 Key Laboratory of Birth Defects and Related Diseases of Women and Children, Sichuan University, Ministry of Education, Chengdu, China; 5 West China School of Medicine, Sichuan University, Chengdu, China; 6 West China School of Pharmacy, Sichuan University, Chengdu, China; 7 West China Biomedical Big Data Center, West China Hospital, Sichuan University, Chengdu, China; 8 Department of Epidemiology and Bio‐statistics, School of Public Health, Peking University, Chengdu, China; 9 Research Center of Clinical Epidemiology, Peking University Third Hospital, Chengdu, China; 10 Chinese Evidence-based Medicine Center, West China Hospital, Sichuan University, Chengdu, China

**Keywords:** adverse events, pharmacovigilance, scoping review, TreeScan, vaccine safety

## Abstract

**Background:**

The TreeScan method is an emerging tool for active safety signal surveillance and has been increasingly applied in post-marketing monitoring of pharmaceuticals and vaccines.

**Objective:**

To evaluate methodological developments and application patterns of the TreeScan method.

**Methods:**

A scoping review was conducted by searching Embase, Medline, Cochrane Library, China National Knowledge Infrastructure, Wanfang, VIP, and SinoMed from inception to 16 May 2025. Two researchers independently screened studies and extracted data. Descriptive analyses were performed on study characteristics, methodologies, and application domains. Included studies were categorized as methodological or applied research.

**Results:**

Forty-four articles were included, comprising 13 methodological studies (29.5%) and 31 applied studies (70.5%). Applied studies included drug safety surveillance (n = 10), vaccine safety surveillance (n = 16), and other areas such as drug repurposing and epidemiology (n = 5). Most studies originated from the United States (n = 28) and South Korea (n = 8). The Bernoulli model (43.2%), Poisson model (18.2%), and tree-temporal scan statistic (29.5%) were the most frequently used approaches. Positive controls we1re used in 63.6% of studies, while 36.4% employed within-group controls. Vaccine safety surveillance represented the most common application area, whereas methodological innovations focused on improving statistical performance, controlling confounding, and extending TreeScan to new data structures.

**Conclusion:**

TreeScan research is increasingly application-oriented, particularly in vaccine safety surveillance Recent methodological advances have improved its performance in handling confounding, hierarchical outcomes, and complex data structures. Future research should should prioritize validating newer TreeScan variants across diverse real-world databases and expanding applications beyond safety surveillance.

## Highlights

- The TreeScan methodology is an important and evolving statistical tool for signal detection in pharmacovigilance and vaccinovigilance, with its application scope expanding into areas such as drug repurposing.
- This scoping review identifies that applied research, particularly in vaccine safety surveillance, currently constitutes the primary use of TreeScan, outpacing methodological studies aimed at its refinement.
- Current applications of TreeScan predominantly employ specific probability models (Bernoulli, Poisson) and often rely on positive control strategies for validation, highlighting established practices alongside potential areas for methodological diversification.
- Future studies should aim to further optimize the TreeScan methodology for specific research contexts to enhance its reliability, adaptability, and application across various safety monitoring domains.

## Introduction

Post-marketing drug safety surveillance is a crucial means of safeguarding public health and evaluating medication safety. Traditional monitoring methods primarily rely on passive surveillance through spontaneous reporting systems. While these methods can detect adverse events (AEs) to some extent, they are limited by underreporting, duplicate reports, and selective reporting, often leading to delayed or even undetected AE signals (Pariente et al., 2007; Huang et al., 2014). With the advent of the information age, the widespread application of electronic healthcare data has promoted the development of active surveillance models, enhancing the sensitivity and efficiency of signal detection (Arnaud et al., 2017).

The tree-based scan statistic (TreeScan) method is an emerging tool for AE signal data mining applied in post-marketing drug safety surveillance in recent years (Kulldorff et al., 2003). This method is implemented based on longitudinal electronic medical record databases. It constructs a drug-event tree structure, calculates the relative risk of each node, and estimates *P*-values using Monte Carlo simulation for significance testing. Without the need for explicit prior hypotheses, TreeScan can effectively identify potential AE signals, providing a basis for further causal relationship validation of candidate drug-event pairs in pharmaceutical and vaccine research (Kulldorff et al., 2013).

This method was initially proposed by Kulldorff et al., 2003 in 2003 for occupational disease mortality monitoring and was later applied to AE signal mining in 2013 (Kulldorff et al., 2003; Kulldorff et al., 2013). In recent years, the number of international studies on this method and its application fields have continued to increase, and the methodology has been further refined. Therefore, given the broad and heterogeneous nature of TreeScan-related research, a scoping review was conducted to map existing evidence, summarize methodological developments and applications, and identify current research gaps and future directions.

## Methods

This study employed the scoping review methodology and was reported in accordance with the PRISMA extension for scoping reviews (PRISMA-ScR).

### Search strategy

Systematic searches were conducted in Embase (Ovid), Medline (Ovid), Cochrane Library, China Biology Medicine disc (CBM), China National Knowledge Infrastructure (CNKI), Wanfang Database, and VIP Database from inception until 16 May 2025. The reference lists of included articles were also reviewed. The core search terms were “TreeScan” OR “tree-based scan” OR “tree-temporal scan”. The search strategy was adapted according to the specific features of each database. Detailed search strategies and results are provided in Supplementary Table S1.

### Inclusion and exclusion criteria

Studies related to TreeScan were included. The exclusion criteria were as follows: ① Non-Chinese or non-English literature; ② Duplicate publications; ③ Methodological reviews of drug safety research methods; ④ Conference abstracts or literature for which the full text was unavailable.

### Study selection and data extraction

Two researchers independently screened the literature, extracted data, and cross-checked the results. Any disagreements were resolved through consultation with a third researcher. During the literature screening process, titles and abstracts were first reviewed. After excluding clearly irrelevant records, the full texts of potentially relevant articles were retrieved and reviewed to determine final inclusion.

The extracted data primarily included: ① Basic publication information, including title, author, publication year, author affiliation, journal, and country/region; ② Methodology, including statistical methods used and types of controls; ③ Study information, including study type, research content, study population, drug information (generic name, Anatomical Therapeutic Chemical [ATC] code), adverse event information (adverse event name, adverse event code), and database information (name, country).

### Data synthesis

A content analysis approach from qualitative research was adopted. Count data were described using frequencies and percentages. The trend in the number of publications over time, distribution of countries of publication, research institutions, drugs, and databases were analyzed using Excel 2019. Data visualization was performed using R version 4.4.0.

## Results

### Literature search and selection

**FIGURE 1:**
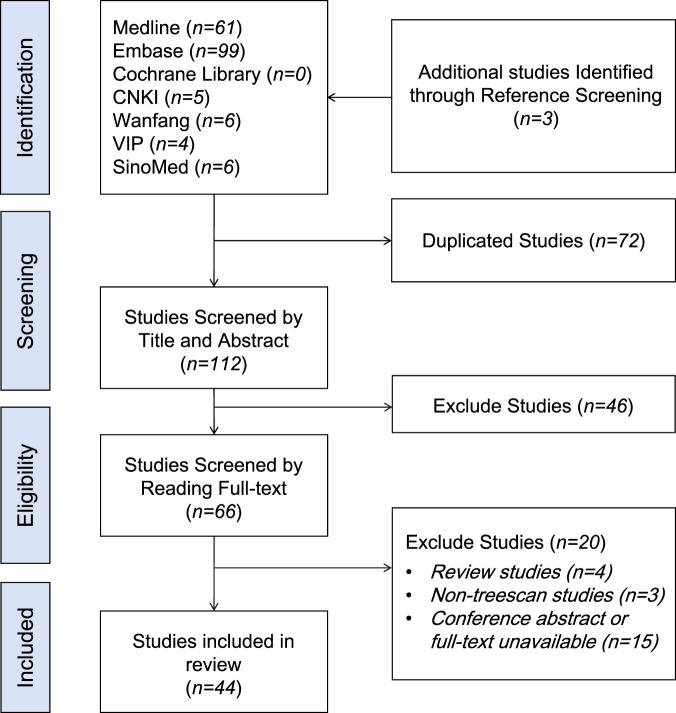
Literature search and selection flowchart.

A total of 184 records were identified through database searches and reference list screening. After removing duplicates and applying the screening process, 44 articles met the inclusion criteria and were included in the analysis. The literature search and selection flow diagram is shown in Figure 1.

### Basic characteristics of included studies

**FIGURE 2:**
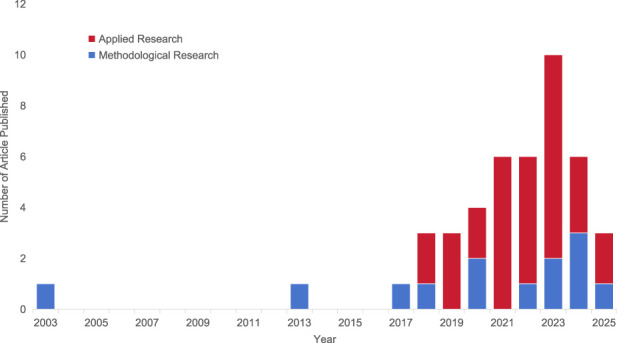
Annual number of TreeScan publications (through May 2025).

Among the 44 included studies, 13 were methodological studies and 31 were application studies. Of the application studies, 10 focused on drug-related AEs, 16 on vaccine-related AEs, and 5 were other types of studies (e.g., drug utilization, drug exposure, and epidemiological studies). The publication years of the included studies ranged from 2003 to 2025, with the majority published after 2017. The year 2023 had the highest number of publications (10 articles) (Figure 2).

The countries of publication were primarily the United States (28 articles, 63.6%) and South Korea (8 articles, 18.2%). The top three institutions by number of publications were Harvard Medical School and Harvard Pilgrim Healthcare Institute (11 articles, 25.0%), Harvard Medical School and Brigham and Women’s Hospital (4 articles, 9.1%), and Sungkyunkwan University (3 articles, 6.8%).

The study populations varied based on research needs. The study duration ranged from 1 year to 29 years. Among the studies reporting the study period, 23 had a duration ≤5 years and 12 had a duration >10 years. The sample sizes of the included studies ranged from a minimum of 4,499 to a maximum of 4,577,434, as shown in Table 1. Detailed basic characteristics of the included studies are provided in Supplementary Table S2.

**TABLE 1:** Basic characteristics of included studies.

Characteristic	Number of studies
Study type	
Methodological research	13
Applied research	31
Country/Region	
United States	28
South Korea	8
China	2
Belgium	2
Taiwan	2
Sweden	1
Australia	1
Institution	
Harvard medical school and harvard pilgrim healthcare institute	11
Harvard medical school and brigham and Women’s hospital	4
Sungkyunkwan university	3
Yonsei university college of medicine	2
University of Texas medical branch	2
Ewha womans university	2
GlaxoSmithKline	2
Sichuan university	2
Study period (years)	
<5	21
5–10	9
≥10	11
NI	3
Sample size (n)	
<100,000	16
100,000–1,000,000	18
≥1,000,000	10

### Methodology of included studies

The identified methodological studies primarily focused on three areas: improving statistical performance, addressing confounding and control selection, and extending TreeScan to new analytical settings. Representative studies within each theme are summarized below.

The tree-based scan statistic was first proposed by Kulldorff et al., 2003, primarily based on the Poisson probability model (Kulldorff et al., 2003). In subsequent methodological studies, 2 studies used the Bernoulli probability model (Wang et al., 2018; Park et al., 2020; Hsieh et al., 2023), 4 studies used the Poisson probability model (Kulldorff et al., 2013; Maro et al., 2017; Lee et al., 2020; Thai et al., 2024), 1 study compared the Bernoulli and Poisson probability models (Suarez et al., 2023a), and the remaining studies proposed new or modified statistical methods based on TreeScan. Among these, 2 studies modified the statistical method based on the hierarchical tree-based scan statistic: Heo et al. (2024) described a method suitable for paired case-control data within a hierarchical tree structure, and Straub et al. (2025) proposed a hierarchical clustering analysis (HCA)-enhanced tree-based scan statistic. Haguinet et al. proposed the self-controlled tree-temporal scan (SCTTS), a tree-temporal scan based on group comparison (BGTTS), and a log-rank based tree-temporal scan (LgRTTS) (Haguinet et al., 2024). Park et al. proposed a tree-based scan statistic for zero-inflated count data (TreeScan-ZIP) and compared it with the existing tree-based scan statistic (Park et al., 2020).

**FIGURE 3:**
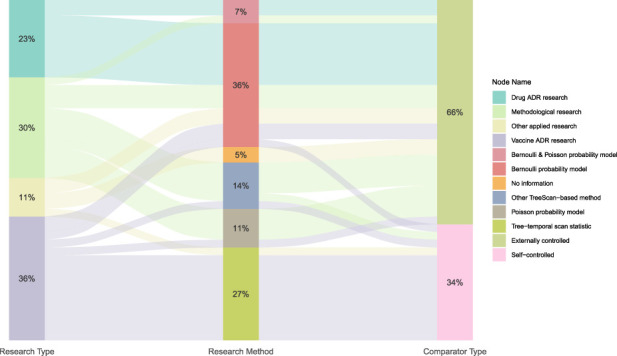
Sankey diagram showing the correspondence between study type, research method, and control type involved drugs, vaccines, and databases.

In drug-related AE studies, 8 studies (80.0%) used the Bernoulli probability model (Schachterle et al., 2019; Wintzell et al., 2020; Fralick et al., 2021; Wang et al., 2021a; Polychronopoulou et al., 2022; Li et al., 2022; Hailong et al., 2023; Kuo et al., 2024), and 2 studies (20.0%) used both the Bernoulli and Poisson probability models (Huybrechts et al., 2021; Suarez et al., 2023b). All studies used other drugs as controls. In vaccine-related AE studies, 3 studies used the Bernoulli probability model (18.8%) (Mahaux et al., 2019; Lee et al., 2022; Lorman et al., 2023a), 1 study used the Poisson probability model (6.3%) (Kim et al., 2020), 11 studies used the tree-temporal scan statistic (68.8%) (Kim et al., 2020), and 1 study used Sequential binomial tree-based scan statistics (6.3%) (Yih et al., 2023). Studies using the tree-temporal scan statistic all employed a self-controlled design. One study using the Bernoulli probability model and one using Sequential binomial tree-based scan statistics also used self-control, while the remaining studies used external controls (other vaccines or non-diseased populations as controls). Among other types of studies, 2 studies on drug exposure and drug utilization used the Bernoulli probability model with case-control and other drug controls, respectively (Wang et al., 2021b; Tan et al., 2024). One epidemiological study used the tree-temporal scan statistic with self-control (Jeong et al., 2025). Two studies did not specify the exact method used (Shiner et al., 2022; Rozema et al., 2024). The correspondence between study type, methodological approach, and control method is shown in the Sankey diagram in Figure 3.

The included studies involved a total of 35 different drugs or vaccines. The top three drugs/vaccines by number of studies were the recombinant zoster vaccine (4 studies), COVID-19 vaccines (3 studies), and sodium valproate (3 studies). Drugs were classified according to the World Health Organization’s Anatomical Therapeutic Chemical (ATC) classification system, resulting in 6 drug classes. The class with the most studies was “Anti-infectives for systemic use,” as shown in Table 2.

**TABLE 2:** Drug classes involved in TreeScan studies.

ATC level 1 code	Drug class	Specific drugs	Number of studies
J	Antiinfective for systemic use	Macrolides, azithromycin, clarithromycin, fluoroquinolones, non-fluoroquinolones, cephalosporins, itraconazole, zoster vaccine, varicella vaccine, pneumococcal vaccines, meningococcal conjugate vaccine, quadrivalent human papillomavirus vaccine, 9-valent human papillomavirus vaccine, influenza vaccines, COVID-19 vaccines, rotavirus vaccine	21
A	Alimentary tract and metabolism	DPP-4 inhibitors, SGLT2 inhibitors, rosiglitazone, pioglitazone, canagliflozin, voglibose, acarbose	7
L	Antineoplastic and immunomodulating agents	BCG vaccine, TNF-α inhibitors, paclitaxel, docetaxel, trastuzumab	4
N	Nervous system	Opioid agonists, buprenorphine, methadone, sodium valproate, lamotrigine, topiramate, GABAergics	4
C	Cardiovascular system	Statins, alpha-blockers, beta-blockers, angiotensin II receptor blockers, calcium channel blockers	3
D	Dermatologicals	Terbinafine	2
M	Musculo-skeletal system	Meloxicam, celecoxib	1

* DPP-4: Dipeptidyl peptidase-4; SGLT2: Sodium-glucose cotransporter-2; TNF-α: Tumor necrosis factor-alpha; GABA: Gamma-aminobutyric acid.

The top three diagnostic coding systems used for identifying AEs were the International Statistical Classification of Diseases and Related Health Problems, 10th Revision (ICD-10), ICD-9, and the World Health Organization Adverse Reaction Terminology (WHO-ART), used in 18, 15, and 5 articles, respectively. Additionally, one article used the Medical Dictionary for Regulatory Activities (MedDRA), one article used the Korean Standard Classification of Diseases, Seventh Revision (KCD-7) codes, and one article used the Multi-Level Clinical Classification Software Refined (CCSR). A total of 33 studies reported statistically significant AE findings using TreeScan. The reported AEs were categorized according to the anatomical chapters of ICD-10; the results are presented in Table 3. Detailed information on exposure-event pairs, including drugs/vaccines, adverse events, databases, study designs, and TreeScan models, is provided in Supplementary Table S2.

**TABLE 3:** Types of adverse reactions involved in TreeScan studies.

Type of adverse reactions	Number of studies
Injury, poisoning and certain other consequences of external causes	21
Symptoms, signs and abnormal clinical and laboratory findings, not elsewhere classified	19
Diseases of the musculoskeletal system and connective tissue	17
Diseases of the skin and subcutaneous tissue	17
Diseases of the digestive system	14
Endocrine, nutritional and metabolic diseases	14
Diseases of the respiratory system	13
Diseases of the circulatory system	13
Diseases of the genitourinary system	12
Certain infectious and parasitic diseases	11
Diseases of the nervous system	9
Diseases of the blood and blood - forming organs and certain disorders involving the immune mechanism	9
Mental and behavioural disorders	6
Factors influencing health status and contact with health services	6
Congenital malformations, deformations and chromosomal abnormalities	5
Diseases of the eye and adnexa	4
Codes for special purposes	4
Neoplasms	3
External causes of morbidity and mortality	2
Pregnancy, childbirth and the puerperium	2
Certain conditions originating in the perinatal period	2
Diseases of the ear and mastoid process	1

Forty studies utilized database data, involving 20 databases from 6 countries or regions. The IBM® MarketScan® Research Databases, the Korea Adverse Event Reporting System (KAERS), and the U.S. Centers for Disease Control and Prevention (CDC)’s Vaccine Safety Datalink (VSD) were the top three databases by number of studies, as shown in Table 4. Furthermore, 3 studies used cohort data (Straub et al., 2025; Shiner et al., 2022; Rozema et al., 2024), and 1 study used data from two Phase III clinical trials (Haguinet et al., 2024).

**TABLE 4:** Databases involved in TreeScan studies.

Database type	Name	Country/Region	Number of studies
Health insurance claims	IBM® MarketScan® research databases	United States	12
	The national health insurance service claims data	South Korea	2
	Optum Clinformatics™ insurance claims database	United States	2
	Taiwan’s national health insurance database (NHID)	Taiwan	1
	20% national sample of medicare claims data	United States	1
	The linked Texas cancer registry (TCR) medicare and surveillance, epidemiology, and end results (SEER) medicare databases	United States	1
Immunization/Vaccine registry	The U.S. Centers for disease control and prevention (CDC)’s vaccine safety datalink (VSD)	United States	4
	Korea disease control and prevention agency vaccination data	South Korea	2
	Taiwan national immunization information system (NIIS)	Taiwan, China	1
Regional health/Population registers	Yinzhou healthcare database	China	2
	Danish population-based registers	Denmark	1
	CDC’s system for enteric disease response, investigation, and coordination (SEDRIC)	United States	1
	Medicare data from New Jersey and Pennsylvania	United States	1
	The national center for health statistics (NCHS) multiple cause of death database	United States	1
Spontaneous reporting	Korea adverse event reporting system (KAERS)	South Korea	5
	GSK batch genealogies and the company’s vaccine spontaneous safety database	Belgium	1
	The korea institute of drug safety & risk management-korea adverse event reporting system database (KIDS-KD)	South Korea	1
Electronic health records	9 HMO plans from HMORN-CERT	United States	1
	The RECOVER PEDSnet EHR	United States	1
Distributed data network	The United States food and drug Administration’s (FDA’s) sentinel system	United States	2
Clinical trial data	Pooled data from ZOE-50 and ZOE-70 trials (phase III RZV vaccine studies)	Global multicenter clinical trial	1
Cohort data	A cohort containing veterans using any VA services	United States	1
	A previously established retrospective cohort that included all VA users with a clinical diagnosis of PTSD	United States	1
	Medicaid MAX/TAF and MarketScan commercial claims mother-child linked pregnancy cohorts	United States	1

## Discussion

### Methodological research

To account for correlations between study subjects and address the issue of increased type I error due to multiple testing, Professor Kulldorff from Harvard University first proposed the TreeScan method in 2003 and applied it to disease surveillance (Kulldorff et al., 2003). This study successfully used the TreeScan method to mine meaningful signals and suggested its potential application in other fields, such as pharmacovigilance. In 2013, Dashevsky et al. (Kulldorff et al., 2013) first applied the TreeScan method to safety signal detection. Their research demonstrated that the TreeScan method is an effective data mining tool for drug safety surveillance, and the statistically significant signals it discovers can serve as candidate drug-event pairs for pharmacoepidemiological studies, awaiting further analysis to validate their causal relationships. In 2017, Maro et al. (2017) published a methodological study reporting on sample size and statistical power calculation for the TreeScan method, providing methodological guidance for researchers designing and optimizing retrospective data mining studies based on the TreeScan method.

Like other safety signal mining methods, the TreeScan method is also susceptible to confounding bias. To address this issue, Wang et al. (2021a) combined the TreeScan method with propensity score methods and employed a new-user cohort study design to mine AE signals. The study evaluated the performance of this approach using simulation experiments. The results showed that this method effectively controlled for confounding factors and maintained a stable type I error rate. It demonstrated good performance in screening and prioritizing potential adverse events.

Existing studies have used simulated data to validate the statistical performance of the TreeScan method. In 2020, Lee et al. (2020) explored potential safety signals for two pneumococcal vaccines in the Korean spontaneous reporting database and compared the signal detection results and performance of four disproportionality methods and the TreeScan method. In a study published in 2020, Park et al. (2020) compared the performance of various signal detection methods, including TreeScan, for mining hierarchical adverse drug event data through simulation experiments. Both studies concluded that TreeScan offers balanced signal detection performance and effectively controls the type I error rate. To address situations where AE databases contain a large number of zero-count cells, Park and Jung (2022) proposed a TreeScan variant suitable for zero-inflated count data within hierarchical structures in a 2022 study. Simulation studies indicated that the new method outperforms existing methods in the presence of excess zeros. In a 2023 study, Suarez et al. (2023a) used simulated data on drug exposure during pregnancy and offspring outcomes to evaluate the statistical performance of the Bernoulli and Poisson probability models. The results suggested that the Poisson model, with its high sensitivity, could be prioritized in this application scenario. The study by Thai et al. (2024) also used simulated data to evaluate the performance of the TreeScan method based on the Poisson probability model (TBSS). The results indicated that with small sample sizes, inaccurate alerting, inflation of type I error, and removal of very rare outcomes for TBSS analysis might occur due to “zero” background counts. Haguinet et al. (2024) used both clinical trial data and simulated data to compare three methods: the self-controlled tree-temporal scan (SCTTS), a tree-temporal scan based on group comparison (BGTTS), and a log-rank based tree-temporal scan (LgRTTS). The results showed that these three methods provide complementary analytical strategies for safety signal detection in clinical trials of pharmaceuticals, suggesting TreeScan can be effectively used for safety analysis in clinical trials. Heo et al. (2024), focusing on the retrospective case-control study design, proposed a TreeScan method for matched case-control data based on the McNemar test, the Wald test from conditional logistic regression, and the multinomial likelihood ratio test. Simulation studies demonstrated that the new method performed better compared to the existing Bernoulli model-based approach.

In a 2025 study, Straub et al. (2025) proposed a hierarchical clustering analysis (HCA)-enhanced TreeScan method and validated it using database data. The results showed promise when defining phenotypes for surveillance and causal inference studies. Hsieh et al. (2023) integrated TreeScan with sequence symmetry analysis (SSA) as a data mining tool to construct a novel classification system that can be used for safety signal mining in real-world longitudinal data.

### Application research

This review included 10 application studies related to drug safety. The data sources for these studies included health insurance claims databases, hospital electronic medical record data, and national electronic health data, with the IBM® MarketScan® Research Databases being the most commonly used. Study populations were not limited to adults but also included special populations such as children and pregnant women. The investigated drugs included anti-infectives, antineoplastic agents, hypoglycemic agents, antipsychotics, and cardiovascular drugs, among others. Study designs included cohort, case-control, and nested case-control studies. All studies employed the Bernoulli probability model and selected other drugs as controls.

This review included 16 application studies related to vaccine safety. Vaccine safety has become a popular application area for the TreeScan method in recent years. Data sources for these studies included health insurance claims databases, active vaccine safety surveillance databases, and spontaneous vaccine adverse event reporting data, covering populations such as adults, the elderly, and children. The studied vaccines included COVID-19 vaccines, meningococcal conjugate vaccines, human papillomavirus vaccines, live attenuated zoster vaccines, and BCG vaccine, among others. Sources of adverse events primarily included diagnostic information (ICD codes) and adverse event reports (MedDRA codes). Most vaccine studies utilized the tree-temporal scan statistic and employed a self-controlled design. This approach not only allows for the investigation of associations between exposure and multiple adverse event outcomes but also, by analyzing the probability of adverse events occurring over different risk periods post-exposure, can identify the specific time points when particular adverse events cluster after exposure or intervention (Sun et al., 2021). These studies not only identified and verified known adverse event signals from various angles but also discovered new signals, providing hypotheses for subsequent validation studies. Notably, one study used the TreeScan method to explore manufacturing-related safety signals. In 2019, Mahaux et al. (2019) used GlaxoSmithKline’s vaccine batch lineage information and spontaneous vaccine safety reporting database, applying the TreeScan method to detect adverse events related to manufacturing. The study suggested that while the method requires further refinement, it can effectively enhance safety signal detection capability and help improve quality issues in the manufacturing process.

Beyond drug safety studies, the included research also involved other drug-related areas such as drug exposure and drug repurposing. A 2020 study by Wang et al. (2021b), using a nested case-control design, employed the TreeScan method to screen for drugs that might delay the progression of neovascular age-related macular degeneration. A 2022 study by Shiner et al. (2022) constructed a mechanistic tree of all U.S. Food and Drug Administration-approved medications and used the tree-based scan statistic to identify medications associated with greater than expected levels of clinically meaningful improvement in PTSD symptoms using electronic health record data from the U.S. Department of Veterans Affairs. Similarly, Rozema et al. (2024) constructed a mechanistic tree of all U.S. Food and Drug Administration-approved medications and used a tree-based scan statistic, TreeScan, to identify medications associated with greater than expected improvements in alcohol consumption. Tan et al., 2024) used tree-based scan statistics to generate repurposing hypotheses for sodium-glucose cotransporter-2 inhibitors (SGLT2i).

The TreeScan method has also been applied in the field of epidemiology. In 2025, Greene et al. (2025) applied tree-temporal scan statistics prospectively to genomic surveillance data with a hierarchical nomenclature for COVID-19 and salmonellosis cases. The study demonstrated that TreeScan assisted in detecting emerging variants and clusters of communicable diseases and in prioritizing them for investigation. Furthermore, other studies have used the TreeScan method to identify patient characteristics associated with selection (Lorman et al., 2023b) or variables included in predictive models (Lin et al., 2023) from electronic health record databases.

Compared with traditional disproportionality methods, such as PRR and ROR, TreeScan offers the advantage of simultaneously evaluating hierarchical outcome structures while effectively controlling for multiple testing. In contrast to sequential monitoring approaches, TreeScan is primarily designed for exploratory signal detection in large healthcare databases rather than real-time surveillance. However, similar to other data mining approaches, TreeScan generates hypothesis signals rather than causal estimates, and its performance is influenced by data structure, coding systems, and study design (Lee et al., 2020; Hailong et al., 2023).

### Limitations and future perspectives

This scoping review has several limitations. First, we only searched Chinese and English databases, which might not cover all studies utilizing the TreeScan method. In addition, conference abstracts were excluded because they often provide insufficient methodological details for data extraction and synthesis. Consequently, some emerging methodological developments may not have been captured in this review. Second, we did not assess the quality or risk of bias of the included studies, as this is a scoping review primarily aimed at describing literature characteristics, reported content, and methodological research progress to illustrate the application and development of TreeScan. Additionally, due to limitations in literature access, studies with negative results might not have been published in journals, potentially leading to publication bias.

In future research, selecting appropriate methods based on the study population and data type is crucial, and methods can be optimized specifically for different data types. Further methodological development could focus on optimizing TreeScan for different healthcare data structures and validating newly developed approaches in diverse real-world databases. Studies could focus on known AEs associated with the drug or vaccine under investigation to validate the statistical power of the method. If the study design selects other populations as controls, methods like propensity score matching can be used to control for bias. Beyond drug and vaccine safety surveillance, future studies could explore the application of TreeScan in broader fields, such as disease surveillance, epidemiology, and drug repurposing research.

## Conclusion

TreeScan is a promising and increasingly utilized approach for safety signal detection, with particular strengths in the analysis of hierarchical outcome data. Continued methodological refinement and validation across diverse real-world settings will further clarify its role in active surveillance of drugs (or vaccines).

## Data Availability

The original contributions presented in the study are included in the article/Supplementary Material, further inquiries can be directed to the corresponding author.
