# Supplementary material for: Mapping the applications and methodological characteristics of the TreeScan method in pharmacovigilance and beyond: a scoping review of 44 studies
Source: Front Pharmacol. 2026 Jul 24;17:1905426. doi: 10.3389/fphar.2026.1905426 (PMC13449071; doi:10.3389/fphar.2026.1905426)
Supplement: Supplementary file 1 [file Supplementaryfile1.docx]

**Supplementary Table 1.** Literature search strategy

| **Databases** | **Search strategy** |
| --- | --- |
| CNKI | (篇关摘=树状扫描统计量)资源范围:总库;中英文扩展 |
| Wanfang | (题名或关键词:(树状扫描统计量)) |
| VIP | 题名或关键词=树状扫描统计量 |
| SinoMed | (“树状扫描统计量”[常用字段:智能]) |
| Medline | "TreeScan"[ All Fields] OR " tree-based scan'[All Ficlds ] OR" tree-temporal scan"[ AllFiclds] |
| Embase | ("TreeScan" or "tree-based scan" or tree-temporal scan").tw |
| Cochrane Library | ("TreeScan" OR " tree-based scan" OR " tree-temporal scan"):ti,ab,kw |
